# Distinct activation mechanisms trigger the trypanocidal activity of DNA damaging prodrugs

**DOI:** 10.1111/mmi.13767

**Published:** 2017-08-31

**Authors:** Emma Louise Meredith, Ambika Kumar, Aya Konno, Joanna Szular, Sam Alsford, Karin Seifert, David Horn, Shane R. Wilkinson

**Affiliations:** ^1^ School of Biological and Chemical Sciences Queen Mary University of London, Mile End Road London E1 4NS UK; ^2^ Department of Infectious and Tropical Diseases London School of Hygiene and Tropical Medicine, Keppel Street London UK; ^3^ The Wellcome Trust Centre for Anti‐Infectives Research, School of Life Sciences University of Dundee Dundee UK

## Abstract

Quinone‐based compounds have been exploited to treat infectious diseases and cancer, with such chemicals often functioning as inhibitors of key metabolic pathways or as prodrugs. Here, we screened an aziridinyl 1,4‐benzoquinone (ABQ) library against the causative agents of trypanosomiasis, and cutaneous leishmaniasis, identifying several potent structures that exhibited EC_50_ values of <100 nM. However, these compounds also displayed significant toxicity towards mammalian cells indicating that they are not suitable therapies for systemic infections. Using anti‐*T*. *brucei* ABQs as chemical probes, we demonstrated that these exhibit different trypanocidal modes of action. Many functioned as type I nitroreductase (TbNTR) or cytochrome P450 reductase (TbCPR) dependent prodrugs that, following activation, generate metabolites which promote DNA damage, specifically interstrand crosslinks (ICLs). Trypanosomes lacking TbSNM1, a nuclease that specifically repairs ICLs, are hypersensitive to most ABQ prodrugs, a phenotype exacerbated in cells also engineered to express elevated levels of TbNTR or TbCPR. In contrast, ABQs that contain substituent groups on the biologically active aziridine do not function as TbNTR or TbCPR‐activated prodrugs and do not promote DNA damage. By unravelling how ABQs mediate their activities, features that facilitate the desired anti‐parasitic growth inhibitory effects could be incorporated into new, safer compounds targeting these neglected tropical diseases.

## Introduction

The protozoan parasites *Trypanosoma brucei*, *T*. *cruzi* and various *Leishmania* species are the etiological agents of human African trypanosomiasis (HAT), Chagas disease and Leishmaniasis, respectively. Spread by the hematophagous habits of insect vectors, these pathogens cause more than 55,000 deaths per year and are prevalent in many regions of the world least able to deal with the associated economic burden (http://www.dndi.org/diseases-projects/). Implementation of new surveillance and treatment programmes in conjunction with improved housing and vector control strategies has resulted in a dramatic reduction in disease prevalence. For example, the number of new cases of HAT has fallen from an estimated peak of 450,000 in 1997 to below 20,000 in 2014 while Chagas disease has been eliminated from Chile, Uruguay and several regions of Argentina and Brazil (Barrett, 2006; Schofield *et al*., 2006; WHO, 2014). The success of such strategies has led to the World Health Organization (WHO) aiming to eliminate HAT as a public health problem by 2020 (WHO, 2013).

Currently, drugs represent the only viable option to combat trypanosomal and leishmanial infections although their use is problematic. They often require supervision for administration, can be costly, have limited efficacy and may cause significant toxicity. Additionally, drug resistance is beginning to further limit the efficacy of the available chemotherapeutic arsenal, with antimonials no longer recommended as a first line treatment for leishmaniasis on the Indian sub‐continent (Wilkinson and Kelly, 2009; Barrett and Croft, 2012; Alsford *et al*., 2013; Perry *et al*., 2015). In the case of HAT and Chagas disease, the front line treatments involve nitroheterocyclic‐based prodrugs with nifurtimox or benznidazole monotherapies used to target *T*. *cruzi* while nifurtimox, in combination with difluoromethylornithine (DFMO), is used against a form of HAT prevalent throughout West and Central Africa (Priotto *et al*., 2009; Yun *et al*., 2010; Wilkinson *et al*., 2011). To mediate their trypanocidal effects, nifurtimox and benznidazole undergo an activation reaction catalysed by an FMN‐containing, mitochondrial type I nitroreductase (NTR), generating cytotoxic reduction products (Wilkinson *et al*., 2008; Hall *et al*., 2011; Hall and Wilkinson, 2012). For nifurtimox, this results in the production of an unsaturated open chain nitrile while benznidazole is processed to glyoxal *via* a series of highly reactive intermediates. As type I NTRs are expressed by some unicellular eukaryotes (including trypanosomes and *Leishmania*) but not by metazoan organisms, the bioreductive activity of this enzyme has been exploited to develop a series of novel antiparasitic nitroaromatic‐ and benzoquinone‐based prodrugs (Wilkinson *et al*., 2011; Patterson and Wyllie, 2014). Several of these display significant potency against *T*. *brucei in vitro* while exhibiting little or no toxicity towards cultured mammalian cells.

Quinones represent a class of organic compounds that contain two carbonyl groups attached to a six membered carbocyclic backbone. They are ubiquitous in nature, functioning in various oxidoreductase cascades, with some natural and synthetic variants being of pharmacological interest. In the latter case, many quinone‐based agents often function as prodrugs and must undergo activation before they can mediate their cytotoxic effects, reactions catalysed by quinone oxidoreductases (McKeown *et al*., 2007; Siegel *et al*., 2012). Based on oxygen‐sensitivity, such enzymes can be divided into two groups. Oxygen‐sensitive quinone oxidoreductases, such as NADH cytochrome b_5_ reductase and cytochrome P450 reductase (CPR), can mediate the 1*e^–^* reduction of one of the quinone's carbonyl oxygens to form an unstable semiquinone radical that, under hypoxic conditions, can be further reduced to the hydroquinone derivative (Powis, 1989; O'Brien, 1991). However, in the presence of oxygen, the semiquinone radical can undergo futile cycling, generating superoxide anions and regeneration of the parent compound. In contrast, oxygen‐insensitive quinone oxidoreductases, such as NAD(P)H quinone oxidoreductase 1 (NQO1), catalyse the simultaneous 1*e^–^* reduction of both the quinone's carbonyl oxygens to form the hydroquinone directly (Ernster *et al*., 1962; Iyanagi and Yamazaki, 1970; Siegel *et al*., 2012). Dependent on structural context, this conversion can result in stable pharmacologically active products, as is the case for the benzoquinone ansamycin antibiotics, or unstable metabolites that undergo further rearrangement before exerting their toxic effects, as seen with the antitumor agents mitomycin A and β‐lapachone (Siegel *et al*., 2012).

Due to their favorable redox and electrochemical properties, compounds containing a quinone pharmacophore represent attractive scaffolds for antimicrobial drug development (Pinto and de Castro, 2009; Beena and Rawat, 2013). Screening against trypanosomes has resulted in the identification of various natural and synthetic lead structures postulated to function as prodrugs promoting oxidative stress within the parasite or through formation of toxic bioreductive products (Henderson *et al*., 1988; Kubata *et al*., 2002; Hoet *et al*., 2004; Pinto and de Castro, 2009; Ramos *et al*., 2009; Garavaglia *et al*., 2010; Hall *et al*., 2012). Using recombinant *T*. *brucei* that lack TbSNM1, a DNA repair enzyme that specifically fixes interstrand crosslinks (ICLs) (Sullivan *et al*., 2015), while expressing elevated levels of oxygen‐sensitive (TbCPR) or –insensitive (TbNTR) quinone oxidoreductases, we demonstrate that many aziridinyl 1,4‐benzoquinones (ABQs) possess significant potency towards trypanosomatid parasites, with different structures undergoing distinct activation mechanisms to generate metabolites that subsequently promote DNA damage within the parasite nucleus.

## Results

### Antiparasitic activity of aziridinyl benzoquinones

Compounds containing an ABQ core display potent anticancer properties particularly against cells where NADPH quinone oxidoreductase 1 (NQO1) expression is upregulated (Lin *et al*., 1972; Lusthof *et al*., 1989; Dehn *et al*., 2004). Recently, the antiparasitic activities of such compounds towards *Plasmodium falciparum* and *T*. *brucei* has been investigated with several potential lead structures identified (Grellier *et al*., 2010; Hall *et al*., 2012). Here, we have expanded on these initial trypanosomal screens to evaluate the growth inhibitory properties of a larger ABQ library against *T*. *brucei*, *T*. *cruzi* and *L*. *major* (Table 1). Out of the 34 compounds tested, 20, 14 and 16 had no effect on *T*. *brucei*, *T*. *cruzi* and *L*. *major* growth, respectively, at concentrations of up to 10 µM. These were not analysed further. For the remaining compounds, RH1 exhibited potencies, expressed as the compound concentration that inhibits cell growth by 50% (EC_50_), of <100 nM towards bloodstream form *T*. *brucei*, with a further five (DZQ, ABQ3, ABQ6, TZQ and ABQ22) exhibiting moderate potency towards this parasite (EC_50_ values of 100–500 nM). Against *T*. *cruzi* epimastigotes, DZQ, TZQ and RH1 were highly active, yielding EC_50_ values <10 nM, with others displaying high (EC_50_ values between 10 and 100 nM; MeDZQ, ABQ22 and ABQ24) or moderate (EC_50_ values between 100 and 500 nM; ABQ4, ABQ6 and ABQ14) growth inhibitory effects. When tested against *L*. *major* promastigotes, TZQ exhibited an EC_50_ of <10 nM, with three other compounds (DZQ, RH1 and ABQ25) exhibiting high potency and eight others yielding moderate potency. Of all the ABQs analysed, RH1 was the most potent agent tested against all three parasites, yielding EC_50_ values of 19 ± 1, 3 ± 1 and 68 ± 1 nM against *T*. *brucei*, *T*. *cruzi* and *L*. *major*, respectively.

**Table 1:** Potency of aziridinyl benzoquinones.

	*T*. *brucei* ^a^		*T*. *cruzi*		*L*. *major*		THP‐1^b^
Compound	EC_50_ (nM)	SI	EC_50_ (nM)	SI	EC_50_ (nM)	SI	EC_50_ (nM)
Nifurtimox	2700 ± 100	>37	2600 ± 400	>38	6280 ± 40	>16	>100000
ABQ7, 9–12, 16–19, 28, 31, 32, 34	>10000	–	>10000		>10000		–
DZQ	272 ± 1	1	5 ± 1	36	60 ± 1	3	181 ± 5
MeDZQ	698 ± 57	1	55 ± 2	16	560 ± 139	2	885 ± 23
ABQ3^c^	400 ± 30	–	6200 ± 1160	–	199 ± 4	–	–
ABQ4	1180 ± 160	–	106 ± 25	–	663 ± 6	–	–
RH1	19 ± 1	<5	3 ± 1	<33	68 ± 1	<1	<100
ABQ6	283 ± 41	2	205 ± 53	5	664 ± 2	2	1067 ± 159
ABQ8	670 ± 30	4	1300 ± 108	2	1870 ± 184	2	2951± 770
ABQ13	>10000	–	5550 ± 240	–	>10000	–	–
ABQ14	>10000	–	445 ± 30	–	2270 ± 17	–	–
ABQ15	>10000	–	3310 ± 200	–	>10000	–	–
AZQ	8233 ± 272	–	6000 ± 283	–	>10000	–	–
TZQ	179 ± 1	<1	3 ± 1	<33	9 ± 1	<11	<100
ABQ22	148 ± 1	1	55 ± 2	3	218 ± 20	1	138 ± 25
ABQ23	2300 ± 60	<1	2180 ± 670	<1	348 ± 166	1	<400
ABQ24	1470 ± 120	<1	37 ± 1	<11	473 ± 123	<1	<400
ABQ25	>10000	–	–	–	80 ± 6	–	–
ABQ26	>10000	–	8638 ± 736	–	2360 ± 230	–	–
ABQ27	>10000	–	620 ± 180	–	210 ± 10	–	–
ABQ29	–	–	975 ± 25	–	175 ± 5	–	–
ABQ30	7400 ± 190	<1	980 ± 25	4	174 ± 10	22	3750 ± 320
ABQ33	>10000	–	>10000	–	8157 ± 80	–	–

Data represent the EC_50_ values of various ABQs towards bloodstream form *T*. *brucei*, *T*. *cruzi* epimastigotes, *L*. *major* promastigotes and differentiated THP‐1 cells. All values are means ± standard deviation of four (parasites) or three (mammalian cells) independent experiments.

**a.** Activity of DZQ, MeDZQ, RH1, TZQ and ABQ's 6–9, 14, 15, 20, 22 against *T*. *brucei* was previously reported (Hall *et al*., 2012).

**b.** Differentiated THP‐1 EC_50_ value towards nifurtimox taken from (Voak *et al*., 2013). The Selectivity Index (SI) of certain compounds (fold difference in EC_50_ values of the THP‐1 line relative to parasite) is noted.

**c.** ABQ3 was unstable with its potency diminishing following repeated freeze/thawing.

### Cytotoxicity against mammalian cells

To evaluate whether those ABQs that displayed activity against the three trypanosomatid parasites exhibited toxicity to mammalian cells, their inhibitory properties against differentiated THP‐1 cells was determined (Table 1). For all structures tested, an *in vitro* toxicity was observed (EC_50_ values <10 µM), with two compounds, including RH1, being extremely toxic and yielding EC_50_ values of <100 nM. Comparison of the mammalian toxicity and antiparasitic EC_50_ data allowed a crude measure of each agent's selectivity (the Selectivity Index or SI) toward the pathogen. In many cases, the ABQs displayed a higher potency against THP‐1 cells relative to the parasite, resulting in a SI value <1. Of those that did display preferential activity against the parasite, the observed selective toxicity of most was equivalent to that seen with nifurtimox, an agent whose use in humans is problematic. The toxicity of these compounds towards cultured mammalian cells precluded any attempt to establish the potency of these structures against the intracellular amastigote forms of *T*. *cruzi* and *L*. *major*.

As THP‐1 cells are an immortal human monocyte line and ABQs have potent activity against cancerous cells (Tan *et al*., 1984; Lee *et al*., 1986; Moore *et al*., 1997), a series of screens using a selected quinone series on non‐cancerous mouse peritoneal macrophages were performed. For DZQ, RH1, TZQ and ABQ22, substantial toxicity towards this line was observed with the compounds generating EC_50_ values of 255, <100, <100 and 120 nM, respectively. This clearly demonstrates that ABQs are toxic to primary cells, thus supporting our THP‐1 findings. In light of the mammalian toxicity issues, our focus on ABQs switched from exploring them as potential treatments for infectious diseases to using them as tools to dissect trypanosomal pro‐drug activation pathways and the structure‐activity relationships among this class of compound.

### Different trypanosomal mechanisms activate aziridinyl benzoquinone prodrugs

**Figure 1:**
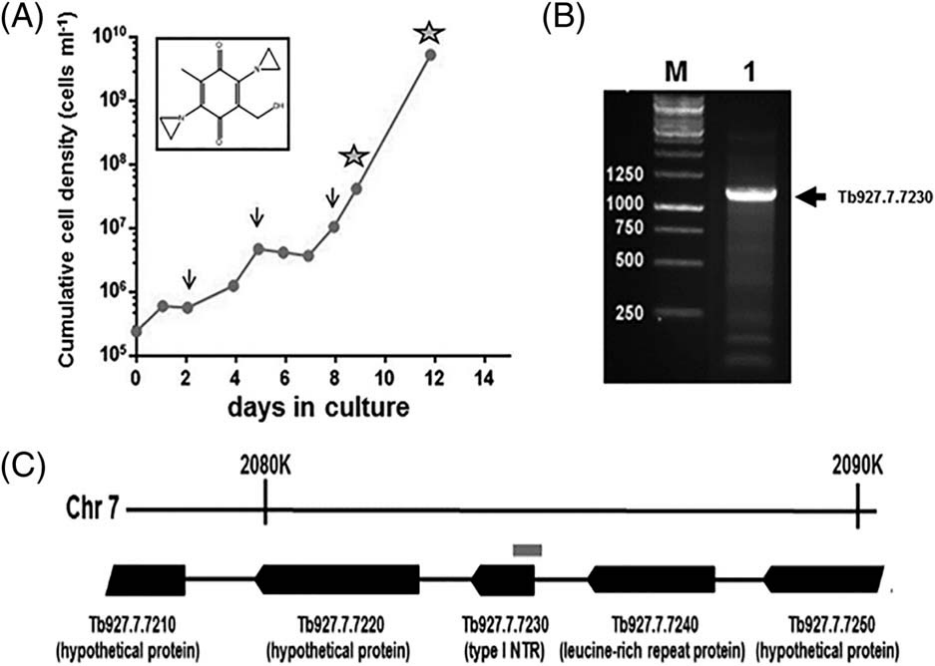
Screening for RH1 resistant determinants using a genome‐scale *T*. *brucei* RNAi library. A. The bloodstream form *T*. *brucei* RNAi library (Alsford *et al*., 2012) was co‐treated with RH1 (30 nM) and tetracycline (1 µg ml^−1^), the latter to induce RNAi, with the cumulative cell growth of the culture followed. The arrows correspond to culture dilution and addition of fresh RH1 (30 nM) and tetracycline (1 µg ml^−1^). Genomic DNA (as indicated by stars) was extracted from parasites at days 9 and 12. Insert shows structure of RH1. B. Amplification of the RNAi target from the RH1 screen using genomic DNA extracted on day 9 of selection and the LIB2f (TAGCCCCTCGAGGGCCAGT) and LIB2r (GGAATTCGATATCAAGCTTGGC) primers (lane 1) produced a major fragment mapping to the type I nitroreductase (Tb*ntr*) locus (gene id Tb927.7.7230 on the TriTrypDB; http://tritrypdb.org/tritrypdb/). M indicates a size marker in bp. C. Genetic map (black boxes represent protein‐coding sequences) of the Tb*ntr* locus, indicating the location of the major RNAi target fragment (grey box) recovered from the library following RH1‐selection.

The application of genome‐scale RNAi screens has helped elucidate the trypanocidal mechanism of action of a number of agents and identify how the affected pathways may impinge on drug resistance (Alsford *et al*., 2012, 2013). As an initial step to understanding how ABQs mediate their antiparasitic activities, a loss‐of‐function screen was conducted on bloodstream form *T*. *brucei*, using RH1 as the selective agent (Fig. 1A). Treatment of the parasite RNAi library with this compound resulted in reduced *T*. *brucei* growth over the first week followed by the outgrowth of an RH1‐resistant population. Genomic DNA was extracted from the RH1‐selected cells and RNAi targets were amplified, from which a single ∼1.1 kbp fragment was identified as the major amplicon (Fig. 1B). Sequence analysis and comparison against the reference genome database revealed that this RH1‐resistance‐associated fragment mapped to a region encompassing the 5′ untranslated region and 5′ coding sequence of the open reading frame (Gene ID: Tb927.7.7230 on TriTrypDB ‐ http://tritrypdb.org/tritrypdb/) for the type I nitroreductase, TbNTR (Fig. 1C). This was not unexpected given that this protein had been previously associated with the activation of trypanocidal nitroheterocyclic‐based prodrugs, nifurtimox and benznidazole (Wilkinson *et al*., 2008; Baker *et al*., 2011; Hall *et al*., 2011; Hall and Wilkinson, 2012) and postulated to function as a NADH dependent quinone oxidoreductase (Wilkinson *et al*., 2008; Alsford *et al*., 2012; Hall *et al*., 2012).

**Figure 2:**
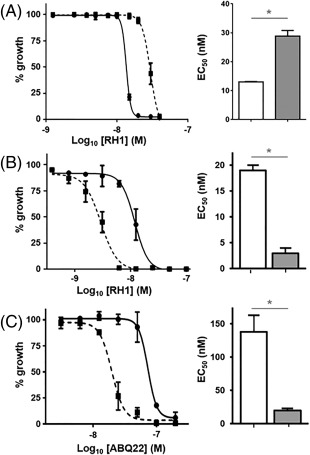
Susceptibility of *T*. *brucei* expressing altered levels of activator to RH1 or ABQ22. A. Dose response curve of wild type (solid line) and Tb*ntr* heterozygous (dotted line) *T*. *brucei* to RH1. The extrapolated EC_50_ values (in nM) of wild type (white bar) and Tb*ntr* heterozygous (grey bar) *T*. *brucei* to RH1 are shown. B. Dose response curve of wild type (solid line) and TbNTR overexpressing (dotted line) *T*. *brucei* to RH1. The EC_50_ values (in nM) of wild type (white bar) and TbNTR overexpressing (grey bar) *T*. *brucei* to RH1 are shown. C. Dose response curve of wild type (solid line) and TbCPR2 overexpressing (dotted line) *T*. *brucei* to ABQ22. The extrapolated EC_50_ values (in nM) of wild type (white bar) and TbCPR2 overexpressing (grey bar) *T*. *brucei* to RH1 are shown. All data are means for experiments performed in quadruplicate ± standard deviation. The asterisk indicates significant differences in susceptibility (*P <* 0.01) between wild type and genetically modified cells to RH1, as assessed by Student's *t* test (GraphPad Software).

To conclusively show that TbNTR is the key activator of ABQs, we evaluated the susceptibility of a *T*. *brucei* Tb*ntr* heterozygote line to RH1. These recombinant cells displayed an EC_50_ approximately 2.5‐fold greater than that observed using wild type parasites (Fig. 2A) confirming that reduction of TbNTR expression through loss of one of the alleles encoding for this oxidoreductase is sufficient to generate resistance to RH1. As reduction of TbNTR activity leads to RH1 resistance, gain of function *via* over expression of the oxidoreductase should have the converse effect, generating cells that are more susceptible to the ABQ. To determine if this was the case, the quinone‐sensitivity of a *T*. *brucei* line engineered to express elevated levels of the enzyme was evaluated. In agreement with previous findings, parasites over expressing TbNTR were hypersensitive (approximately fourfold) to RH1 as compared to controls (Fig. 2B).

To determine whether TbNTR plays a role in activating other ABQs, the susceptibility of TbNTR over expressing parasites towards a selected group of compounds, was tested (Table 2). Out of the additional compounds screened, three (DZQ, MeDZQ and TZQ) showed the same hypersensitivity profile as RH1, with TbNTR over expressing *T*. *brucei* being up to 3.5‐fold more susceptible to the aziridinyl agents than controls. In contrast, ABQ22–24 and 30 did not follow this pattern, with the TbNTR over expressers exhibiting an EC_50_ equivalent to that observed against control cells.

**Table 2:** Growth inhibitory effect of ABQs towards *T*. *brucei* expressing elevated levels of potential activators.

	EC_50_ (nM)		
Compound^a^	Control	Tb*ntr^*++*^*	Tb*cpr2^*++*^*
DFMO	27500 ± 108	30720 ± 1330 (1.12)	33250 ± 5123 (1.21)
Nifurtimox	2980 ± 30	315 ± 15 (0.11^*^)	–
DZQ	318 ± 38	90 ± 3 (0.28^*^)	–
MeDZQ	740 ± 18	115 ± 3 (0.16^*^)	–
RH1	19 ± 1	3 ± 1 (0.16^*^)	20 ± 3 (1.05)
TZQ	225 ± 26	60 ± 9 (0.27^*^)	284 ± 13 (1.26)
ABQ22	138 ± 25	148 ± 9 (1.07)	20 ± 3 (0.14^**^)
ABQ23	2935 ± 420	3315 ± 540 (1.13)	3556 ± 96 (1.21)
ABQ24	1470 ± 120	2090 ± 160 (1.42)	1698 ± 23 (1.16)
ABQ30	7400 ± 190	8670 ± 1670 (1.12)	–

Data represent the EC_50_ values of parasites expressing wild type (control), elevated levels of TbNTR (Tb*ntr* ^++^) or TbCPR2 (Tb*cpr2* ^++^). All values are means ± standard deviation of four independent experiments. The fold difference in EC_50_ values of the over expressing lines relative to control is given in parentheses. Parasites over expressing TbCPR3 behaved similarly to those expressing elevated levels of TbCPR2.

**a.** Trypanocidal activity of DZQ MeDZQ, RH1, ABQ6, TZQ and ABQ22 towards *T*. *brucei* cells over expressing Tb*ntr* was previously reported (Hall *et al*., 2012).

*The difference in susceptibility of wild type and Tb*ntr* ^++^ lines to nifurtimox, DZQ, MeDZQ, RH1 and TZQ was statistically significant (*P <* 0.01), as assessed by Student's *t* test.

**The difference in susceptibility of wild type and Tb*cpr* ^++^ lines to ABQ22 was statistically significant (*P <* 0.01), as assessed by Student's *t* test.

Dependent on backbone configuration, the carbonyl oxygen atoms in a quinone can undergo 1 or 2*e^–^* reduction, with the former reaction carried out by a range of ubiquitous flavoproteins, including CPR, while the latter is catalysed by enzymes, such as NQO1 and type I NTRs. To determine whether the trypanocidal activity displayed by selected ABQs can undergo activation *via* a 1*e^–^* reduction mechanism, the susceptibility of *T*. *brucei* lines engineered to express ectopic copies of the TbCPR2 or 3 (Hall *et al*., 2011) to RH1, TZQ and ABQ22–24 was evaluated (Table 2). With RH1, TZQ, ABQ23 and ABQ24, the TbCPR over expressing lines exhibited EC_50_ values similar to those observed using control cells, indicating that the 1*e^–^* reduction pathway plays no significant role in their activation. When the *T*. *brucei* line expressing elevated levels of TbCPR2 or 3 was tested against ABQ22, the recombinant lines were hypersensitive to this tetra‐aziridinyl agent relative to controls (Fig. 2C).

Based on the above data, the trypanocidal activity of ABQs can be divided into compounds such as RH1 and TZQ that function as TbNTR‐dependent (oxygen‐insensitive quinone oxidoreductase) prodrugs, structures including ABQ22 that function as TbCPR‐dependent (oxygen‐sensitive quinone oxidoreductase) prodrugs and agents such as ABQ23 and 24 that are not activated by either of these two mechanisms: ABQ23 and 24 may still function as prodrugs undergoing activation by an alternative mechanism(s) to that explored here.

### Trypanosomes selected for resistance toward RH1 have reduced NTR expression

**Figure 3:**
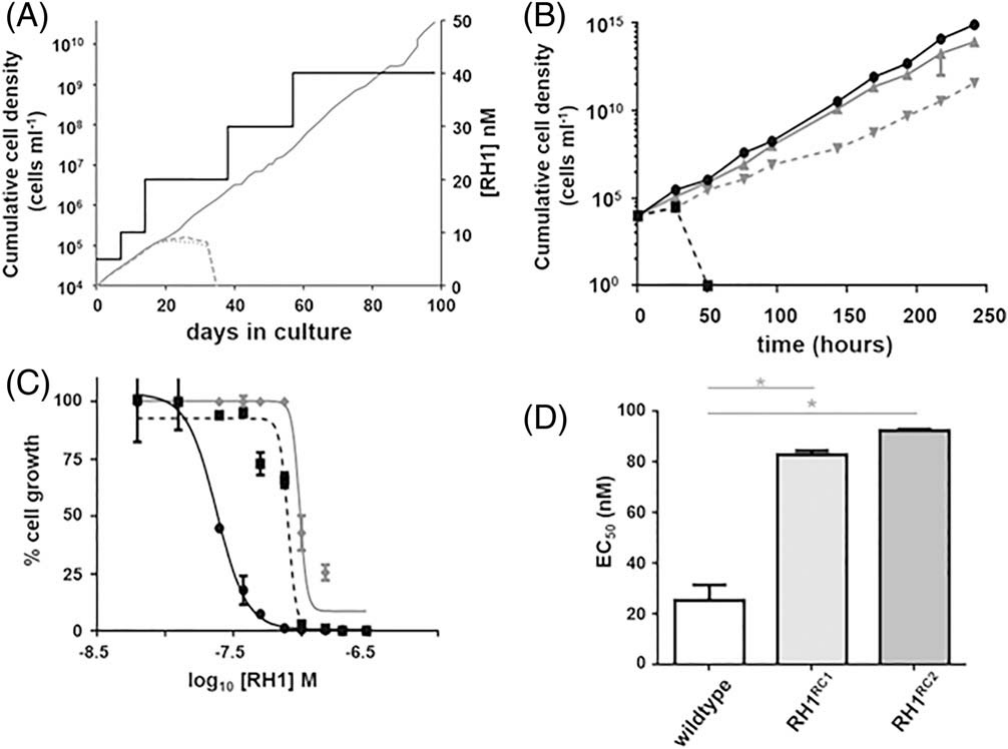
RH1 resistance selection in *T*. *brucei*. A. Selection of RH1 resistant *T*. *brucei* was performed by the stepwise increase of the ABQ (black line). The cumulative cell density of three independent cultures (grey lines) were monitored throughout the selection with only one of these (RH1^R^) generating an outgrowth in medium containing >20 nM RH1. Clones from the RH1^R^ culture were then generated following limiting dilution. B. Cumulative cell density of wild type *T*. *brucei* (black lines) and a clone (RH1^RC1^) derived from RH1^R^ (grey lines) grown in medium lacking (solid lines) or containing 40 nM RH1 (dashed lines). Data points are averages ± standard deviation from experiments performed in quadruplicate. A second RH1^R^ derived clone (RH1^RC2^) analysed in parallel displayed similar growth properties (data not shown). C. Dose response curves of wild type *T*. *brucei* (black solid line), RH1^RC1^ (black dashed line) and RH1^RC2^ (grey solid line) to RH1. All data points are averages from experiments performed in quadruplicate ± standard deviation. D. Susceptibility of *T*. *brucei* to RH1 as judged by the EC_50_ values (in nM). The asterisk indicates significant differences in susceptibility (*P <* 0.001) between wild type and genetically modified cells to RH1, as assessed by Student's *t* test (GraphPad Software).

A further approach to determine how ABQs mediate their trypanocidal activity involved the selection of *T*. *brucei* lines resistant to RH1. Wild type bloodstream form parasites were grown in the presence of RH1 over a 100 day period, starting with a compound concentration that inhibits *T*. *brucei* growth by ∼10% (5 nM) and progressively increasing this to twice the EC_50_ (40 nM) (Fig. 3A). The cumulative cell density of selected cultures was followed throughout the experiment. Of the three replica cultures analysed, two stopped growing when the selective pressure was increased to 20 nM. In contrast, cells in the third culture remained viable in 40 nM RH1 and parasites in this population were cloned by limiting dilution (designated as RH1^RC^). We then evaluated the growth properties of two RH1^RC^ lines (RH1^RC1^ and RH1^RC2^) (Fig. 3B). In the absence of RH1, the doubling time of the two clones was comparable to the parental line (doubling time of ∼ 7.5 hours). When grown in medium containing RH1 (40 nM), the doubling time of the resistant clones increased ∼1.5‐fold (doubling time of ∼ 10.5 hours); by comparison, no growth of wild type *T*. *brucei* was detected after 24 hrs. Next, we determined the susceptibility of the RH1^RC^ lines towards RH1 with this showing that both lines were ∼3.5‐fold less sensitive to the ABQ than wild type (Fig. 3C and D); wild type, RH1^RC1^ and RH1^RC2^ exhibited EC_50_ values of 25 ± 6, 83 ± 2 and 92 ± 1 nM against RH1, respectively. This resistance phenotype was not lost following 100 generations in medium lacking RH1. Indeed, the resistant lines were still ∼4.5‐fold less sensitive to the ABQ than controls; wild type, RH1^RC1^ and RH1^RC2^ exhibited EC_50_ values of 19 ± 1, 86 ± 3 and 91 ± 11 nM against RH1, respectively. When the above susceptibility studies were extended to TbNTR‐activated prodrugs, cross‐resistance towards nifurtimox, benznidazole, megazol, CB1954 and LH17 was observed (Table 3). This phenotype was specific to this class of compound, as the RH1 selected cells were equally sensite to the non TbNTR‐activated compounds, including ABQ22 and other ICL inducing agents, as wild type *T*. *brucei*.

**Table 3:** Susceptibility of RH1 resistant *T*. *brucei* to trypanocidal agents.

	EC_50_ values (nM)		
Compound	Wild type	RH1^RC1^	ratio
*Aziridines*			
Triethylenemelamine	2400 ± 86	2774 ± 64	1.1
ThioTEPA	39423 ± 796	56298 ± 1960	1.4
RH1^a^	25 ± 6	83 ± 2^*^	3.3
ABQ22	197 ± 31	232 ± 27	1.2
CB1954^a^	2161 ± 125	13086 ± 972^*^	6.5
*Nitrogen mustards*			
Mechlorethamine	32063 ± 1021	27203 ± 1288	0.8
Melphalan	8740 ± 200	10430 ± 230	1.2
LH17^a^	5438 ± 444	17998 ± 1106^*^	3.6
*Nitrosoureas*			
Lomustine	16650 ± 443	15463 ± 665	0.9
Semustine	15025 ± 3457	17100 ± 3619	1.1
*Other agents*			
DFMO	16763 ± 2691	22177 ± 383	1.3
Nifurtimox^a^	2700 ± 100	7050 ± 311^*^	2.6
Megazol^a^	166 ± 1	454 ± 4^*^	2.7

Data represent the growth‐inhibitory effect as judged by their EC_50_ values of various agents on BSF *T*. *brucei* wild type and RH1^RC1^ cells. All values are means ± standard deviation from independent experiments preformed in quadruplicate. The ratio represents the fold difference in EC_50_ value between the RH1^RC1^ and wild type lines to a given compound.

**a.** Identifies TbNTR‐activated prodrugs.

*Indicates significant differences in susceptibility (*P <* 0.001) between wild type and RH1^RC1^ lines, as assessed by Student's *t* test (GraphPad Software).

**Figure 4:**
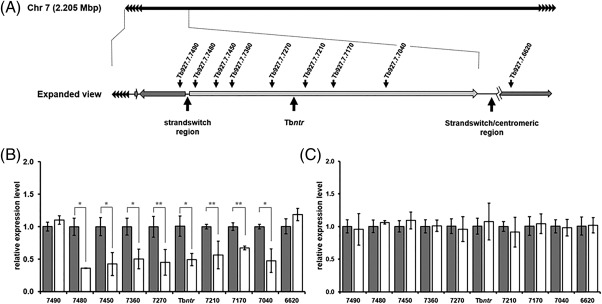
Evaluating the mechanism underlying RH1 resistance in *T*. *brucei*. A. Schematic of *T*. *brucei* chromosome 7 that contains Tb*ntr*. This gene is part of a 200 kbp polycistronic region (see Expanded view) that encodes for ∼60 open reading frames (ORF) (light grey arrowed box) and is approx. 75 kbp downstream from a ‘strandswitch’ region, areas on the trypanosomal chromosome that are postulated to be regions where polycistronic transcription is initiated (Imboden *et al*., 1987; Muhich and Boothroyd, 1988). The position of selected ORFs from the Tb*ntr*‐containing polycistronic transcriptional unit plus the telomeric repeats (black arrow repeats) are shown. Other selected polycistrons on chromosome 7 are also highlighted (dark grey arrowed boxes). B and C. The mRNA levels (panel B) and gene copy number (panel C) of selected ORFs located on chromosome 7 from wild type (grey bar) and RH1^RC1^ (white bar) cells was evaluated by qPCR. This was compared against the expression level/gene copy number of a standardized control (Tb*tert*) and the average fold difference, as judged by 2–(ΔΔC_T_) from reactions performed in triplicate ± standard deviation, plotted as a measure of the relative expression level. The asterisk and double asterisk indicates significant differences in relative mRNA expression levels (*P <*0.01 and *<*0.02, respectively) between the wild type and RH1^RC1^, as assessed by Student's *t* test (GraphPad Software).

Studies aimed at deciphering the RH1 resistance mechanism demonstrated that although the Tb*ntr* copy number was similar and Tb*ntr* gene sequences were identical in the compound selected and wild type lines, the RH1^RC^ cells had a lower (∼50%) Tb*ntr* mRNA expression level (Fig. 4). Intriguingly, when extended to genes expressed in the same polycistron as Tb*ntr*, all the tested ORFs exhibited the same trait in relation to copy number and transcript levels. The observed reduction in gene expression within the Tb*ntr*‐containing polycistron in the RH1 selected cells is specific to this region as additional ORFs present elsewhere in the *T*. *brucei* genome (for example Tb927.7.7490 and Tb927.7.6620, and the genes encoding for the DNA repair enzymes TbSNM1, TbMRE11, TbRAD51, TbXPG, TbEXO1, TbCSB and TbMSH3) are expressed at equivalent levels in RH1^RC^ and control parasites.

### Aziridinyl benzoquinones promote cell cycle arrest and DNA damage in trypanosomes

**Figure 5:**
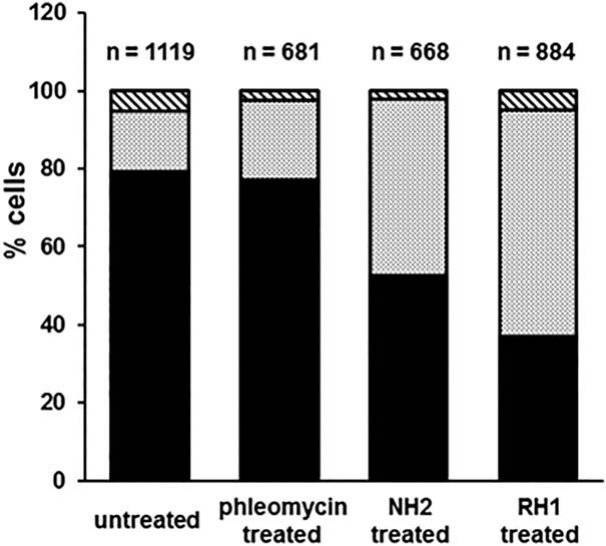
RH1 promotes cell cycle arrest in *T*. *brucei*. The effect of ICL inducing agents (mechlorethamine (HN2) or RH1) on the trypanosome cell cycle was analysed by determining the relative number of nuclear (N) and mitochondrial (K) genomes structures within a single parasite. *T*. *brucei* in the G1/S phase have a 1N1K arrangement (black bar), those in G2/M phase possess a 1N2K ratio (grey bar) while cells displaying a 2N2K profile are in the post‐M stage (hatched bar) (Woodward and Gull, 1990; Siegel *et al*., 2008; Glover and Horn, 2012). The number of cells (n) analysed for each treatment (untreated, phleomycin treated, mechlorethamine (HN2) or RH1) is given.

Aziridinyl benzoquinones mediate their toxicity against mammalian cells through formation of ICLs that promote cell cycle arrest and DNA damage (Kim *et al*., 2004; Dehn *et al*., 2005; Begleiter *et al*., 2007). To determine if this is the case in *T*. *brucei*, parasites at various stages in the cell cycle were identified in asynchronous cultures by staining their nuclear (N) and mitochondrial (known as the kinetoplast (K)) genomes with DAPI; a non‐dividing trypanosome cell usually contains one mitochondrion and hence one kinetoplast. The ratio of these two DNA‐containing structures represents an excellent marker for the trypanosomal cell cycle, with *T*. *brucei* in the G1/S phase having a 1N1K arrangement, those in G2/M phase possessing a 1N2K ratio while cells displaying a 2N2K profile are in the post‐M stage (Woodward and Gull, 1990; Siegel *et al*., 2008; Glover and Horn, 2012). For untreated *T*. *brucei* and cells exposed to phleomycin, most (approximately 80%) were in the G1/S stage of the cell cycle with a smaller percentage (12 to 20%) in G2/M (Fig. 5). In contrast, when trypanosomes were treated with RH1 or mechlorethamine (a well characterized ICL inducing agent), 58% and 45% of the cells displayed a configuration typical of the G2/M phase, respectively, with only 37% and 53% exhibiting a 1N1K DNA staining pattern, respectively. This indicates that treatment of *T*. *brucei* with RH1 (or mechlorethamine) results in a G2/M cell cycle arrest phenotype, potentially as a result of damaged DNA and is consistent with observations made with mammalian cells (Kim *et al*., 2004; Dehn *et al*., 2005).

**Figure 6:**
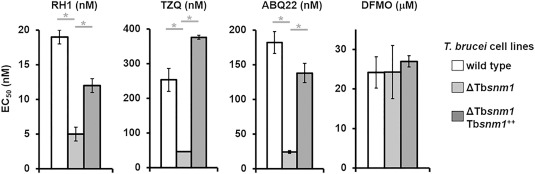
Susceptibility of *T*. *brucei* lines expressing altered levels of TbSNM1 to selected azirindinyl benzoquinones. Growth inhibitory effects, as judged by EC_50_ values, of RH1, TZQ and ABQ22 (all in nM) in the *T*. *brucei* wild type, Tb*snm1* null mutant (ΔTb*snm1)* and Tb*snm1* null mutants expressing an ectopic copy of Tb*snm1* (ΔTb*snm1* Tb*snm1^++^*) lines (Sullivan *et al*., 2015). DFMO (in µM) was used as a non‐DNA damaging agent control. Data are mean values ± standard deviations from experiments performed in quadruplicate. The asterisk indicates significant differences in susceptibility (*P <*0.001) between wild type and ΔTb*snm1* or between ΔTb*snm1* and ΔTb*snm1* Tb*snm1^++^* cells to RH1, as assessed by Student's *t* test (GraphPad Software).

In most cells, ICLs are repaired through the concerted action of a number of complementary and overlapping pathways with enzymes belonging to the SNM1/PSO2 family playing a specific role. In *T*. *brucei*, the enzyme TbSNM1, a member of the SNM1/PSO2 nuclease family, has been shown to play a specific and key role in repairing such damage (Sullivan *et al*., 2015). To evaluate whether deletion of both copies of Tb*snm1* from the *T*. *brucei* genome altered the cells sensitivity to ABQs, the null mutant trypanosomes were grown in the presence of selected trypanocidal quinones and the EC_50_ values for each compound determined (Table 4; Fig. 6). For all the ABQ prodrugs tested, cells lacking the nuclease were up to 6.6‐fold more susceptible to these structures than control parasites. In contrast, assays using ABQ23 and ABQ24, the two ABQs that do not function as TbNTR‐ or TbCPR‐activated prodrugs, and the trypanocidal agents nifurtimox and DFMO, revealed no significant difference in the EC_50_ exhibited by the engineered and control lines.

**Table 4:** Susceptibility of *T*. *brucei* Tb*snm1* null mutants to ABQ compounds.

	EC_50_ (nM)		
Compound	Control	ΔTb*snm1*	Ratio
Nifurtimox	2850 ± 20	2250 ± 090	0.79
DFMO	27500 ± 108	27100 ± 850	0.99
DZQ	157 ± 23	43 ± 2*	0.27
RH1	19 ± 1	5 ± 1*	0.26
TZQ	253 ± 33	46 ± 1*	0.18
ABQ22	138 ± 25	21 ± 5*	0.15
ABQ23	2300 ± 60	2410 ± 160	1.01
ABQ24	1470 ± 120	1130 ± 100	0.77

Data represent the EC_50_ values of selected ABQ compounds towards wild type *T*. *brucei* (control) and *T*. *brucei* Tb*snm1* null mutants (ΔTb*snm1*). All values are means ± standard deviation of four independent experiments. The ration of EC_50_ values between the two parasite lines are given. *Indicates significant differences in susceptibility (*P <* 0.01) between the wild type and ΔTb*snm1* lines, as assessed by Student's *t* test (GraphPad Software).

To conclusively demonstrate that the above altered susceptibility phenotype was solely due to lack of TbSNM1 activity, the sensitivity of null mutant cells expressing an ectopic copy of Tb*snm1* towards DZQ, RH1 and ABQ22 was evaluated (Fig. 6). In all three cases, the TbSNM1‐complemented line generated dose response curves and EC_50_ values distinct from those observed when using TbSNM1 null mutant cells but similar to those obtained using wild type parasites.

### Linking prodrug activation with DNA damage

**Figure 7:**
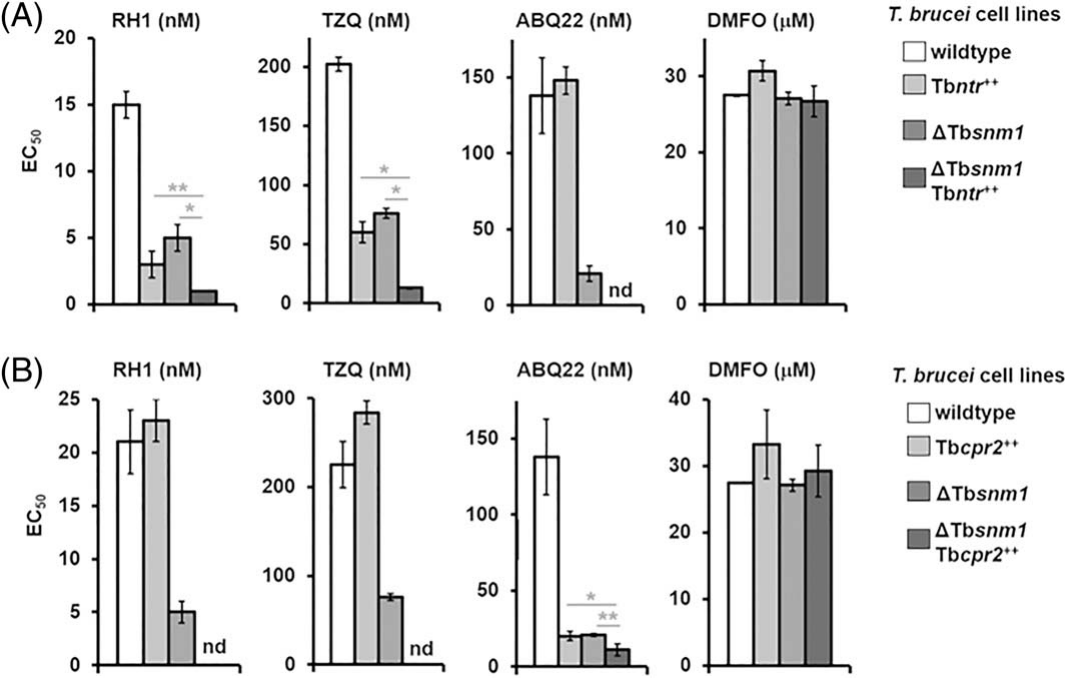
Linking aziridinyl benzoquinone prodrug activation to DNA damage. A. Growth inhibitory effects, as judged by EC_50_ values, of RH1, TZQ, ABQ22 (all in nM) and DFMO (in µM) in the *T*. *brucei* wild type, *T*. *brucei* expressing an ectopic copy of Tb*ntr* (Tb*ntr^++^*), Tb*snm1* null mutant (ΔTb*snm1*) and Tb*snm1* null mutants expressing elevated levels of Tb*ntr* (ΔTb*snm1* Tb*ntr^++^*) lines. The asterisk and double asterisk indicates significant differences in susceptibility (*P <*0.001 and *<*0.03, respectively) between the ΔTb*snm1* Tb*ntr^++^* line relative to the Tb*ntr^++^* or ΔTb*snm1* parasites to RH1 and TZQ, as assessed by Student's *t* test (GraphPad Software). B. Growth inhibitory effects, as judged by EC_50_ values, of RH1, TZQ and ABQ22 (all in nM) in the *T*. *brucei* wild type, *T*. *brucei* expressing an ectopic copy of Tb*cpr2* (Tb*cpr2^++^*), Tb*snm1* null mutant (ΔTb*snm1*) and Tb*snm1* null mutants expressing elevated levels of Tb*cpr2* (ΔTb*snm1* Tb*cpr2^++^*) lines: the latter lines was validated as previous described (Hall *et al*., 2011; Sullivan *et al*., 2015). DMFO (in µM) was used as a non‐DNA damaging agent control. The asterisk and double asterisk indicates significant differences in susceptibility (*P =* 0.01 and *P* < 0.003, respectively) between the ΔTb*snm1* Tb*cpr2^++^* line relative to the Tb*cpr2^++^* or ΔTb*snm1* parasites to ABQ22, as assessed by Student's *t* test (GraphPad Software). Data in panels A and B are mean values ± standard deviations from experiments performed in quadruplicate.

To establish whether there is a link between activation of the trypanocidal ABQs and the TbSNM1‐mediated DNA repair pathway, the susceptibility of Tb*snm1* null‐mutants expressing ectopic copies of Tb*ntr* or Tb*cpr2* towards selected compounds was evaluated (Fig. 7). When these lines were treated with RH1 or TZQ, an increased susceptibility was observed relative to wild type controls (Fig. 7A); Tb*snm1* null‐mutants and *T*. *brucei* over expressing Tb*ntr* were threefold and fivefold more susceptible to RH1, respectively, and 2.7‐ and 3.4‐fold more susceptible to TZQ than wild type *T*. *brucei*. For Tb*snm1* null parasites expressing an ectopic copy of Tb*ntr*, this increase in potency was magnified further, with these cells showing a 15‐fold increase in susceptibility to both compounds as compared against wild type. Extending these studies to investigate the susceptibility of Tb*snm1* null‐mutant parasites engineered to express an ectopic copy of Tb*cpr2* towards ABQ22 yielded similar results (Fig. 7B). When Tb*snm1* null mutants or wild type cells engineered to express an ectopic copy of Tb*cpr2* were treated with ABQ22 a sevenfold increase in susceptibility was observed by both lines compared against controls. The growth inhibitory effect of this compound was further enhanced in trypanosomes that lack TbSNM1 activity while expressing elevated levels of TbCPR2 (Fig. 7B), with these cells being 12.5‐fold more sensitive to ABQ22 than wild type. The above alterations in susceptibilities were specific to the ABQs as all lines tested exhibited equivalent sensitivities to the non‐DNA damaging trypanocidal agent DFMO.

## Discussion

ICLs represent a highly toxic form of DNA damage that blocks processes dependent on DNA strand separation, such as replication and transcription. As rapidly dividing cells are particularly susceptible to this type of lesion, agents that are able to promote ICLs are of interest in the treatment of cancer and infectious diseases (Chen and Hu, 2009; Deans and West, 2011; Wilkinson *et al*., 2011). Here, we report that several ABQs, ICL inducing chemicals originally developed as anti‐cancer agents, can also function as highly potent anti‐trypanosomatid prodrugs with different structures able to undergo activation *via* different pathways. In these cases, activation promotes a type of DNA damage that is repaired by a pathway where SNM1, a nuclease that only functions in ICL repair, plays a key role.

**Figure 8:**
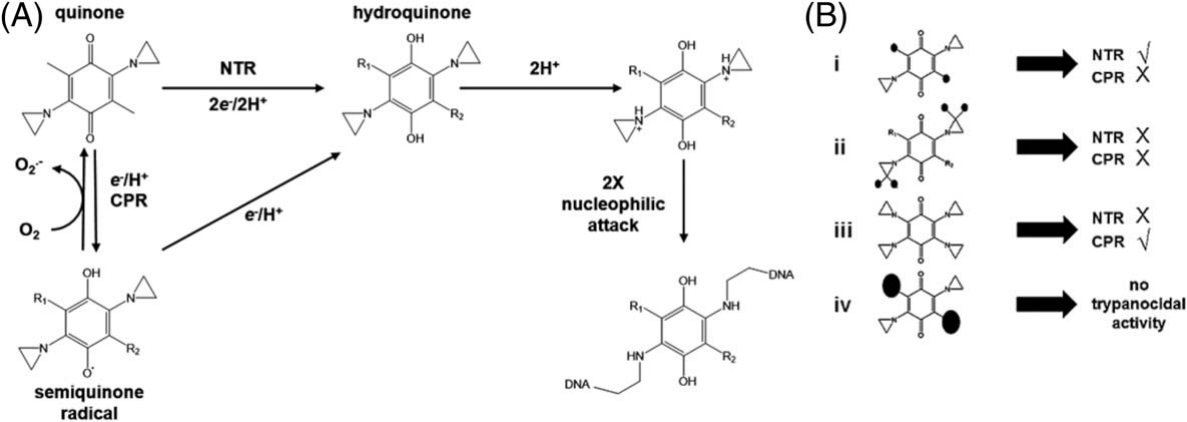
Proposed mechanism of action and trypanocidal structure activity relationships of ICL‐forming ABQs. A. Quinones can be reduced to hydroquinones by two distinct pathways. In one pathway, enzymes such as cytochrome P450 reductase (CPR) mediate the 1*e* ^–^ reduction of the quinone to a semiquinone radical. This then undergoes further reduction to the hydroquinone. In the presence of O_2_, the semiquinone can undergo futile cycling to form $O_{2}^{.-}$ and the parental quinone. In the other system, reduction of the quinone to the hydroquinone occurs directly *via* a 2*e* ^‐^ reduction event as typified by a type I nitroreductase (NTR) activity. Formation of the hydroquinone then facilitates protonation of the nitrogen atoms in the aziridine rings with these electrophilic intermediates undergoing nucleophilic attack (e.g., by DNA), promoting opening of the aziridinyls, formation of aliphatic amine intermediates and alkylation of the target. As ABQs contain two (or more) aziridinyls they can covalently bind the two complementary DNA strands together resulting in an interstrand crosslink. B. ABQs (e.g., DZQ, MeDZQ, RH1 and TZQ) possessing small (e.g., hydrogen, methyl, hydroxymethyl) substituent groups (small solid spheres) at the 3,6 positions on a benzoquinone core are more likely to be activated in *T*. *brucei* by the type I nitroreductase (NTR) but not by cytochrome P450 reductase (CPR) (i). ABQs that contain methyl substituted (small solid spheres) aziridines (e.g., ABQ23, 24 and 30) are not activated in *T*. *brucei* by NTR or CPR (ii). ABQ22, a compound that contains aziridinyl rings at the 3,6 positions on the benzoquinone core, is readily activated within the trypanosome by CPR but not by NTR (iii). ABQs (e.g., ABQ9–19) having bulky substituent groups (large solid spheres) at the 3,6 positions on the benzoquinone ring generally display no significant trypanocidal activity (iv).

Invariably, aziridine‐containing compounds function as prodrugs with activation requiring protonation of the aziridinyl nitrogen. The ease with which this reaction can occur is governed by the compound's chemical composition and how this influences the pKa of the aziridine. For certain structures (e.g., triethylenemelamine, thioTEPA and ‘simple’ ABQs including ABQ11) protonation can readily take place in aqueous solution due to the relatively high pKa of the aziridine within such backbones (Akhtar *et al*., 1975). However, for other agents, the pKa of the heterocycle is comparatively low, therefore preventing the above activation event. To circumnavigate this issue, the aziridine motif can be incorporated into a chemical backbone that also contains a bioreductive moiety, such as a quinone or nitroaromatic. Here, enzymatic reduction of carbonyl oxygen or nitro groups triggers a redistribution of electrons around the associated cyclical structure that can raise the pKa of the aziridinyl ring, and thus facilitating nitrogen protonation (Fig. 8A) (Hargreaves *et al*., 2000).

Using a combination of a whole genome loss‐of‐function screen, selection of resistant lines and/or functional genomic approaches, we have shown that *T*. *brucei* expresses at least two ABQ bioreductive systems. For the eight compounds evaluated as potential prodrugs, half (DZQ, MeDZQ, RH1 and TZQ) were shown to be NTR activated, one (ABQ22) was shown to be dependent on a CPR activity, while the remainder (ABQ23, 24 and 30) were not reduced by any of the systems tested. For those that appeared not to function as prodrugs, this may be due to their structures, as these compounds contain methyl substituted aziridines (Fig. 8B). These alkyl groups may block protonation and thus activation of the aziridinyl nitrogen by lowering this motif's pKa and/or hinder access of oxidoreductases to the carbonyl groups (Lusthof *et al*., 1989; Phillips *et al*., 1999). These possibilities may also account for why other compounds that contain methyl/dimethyl aziridine substitutions (e.g., ABQs 25–30 and 31–34) consistently displayed no or a lower activity against *T*. *brucei* than their non‐substituted counterparts, mirroring observations made in high throughput oncological screens (Kim *et al*., 2016).

For those compounds that do function as trypanocidal prodrugs, the nature of the side chains found at the 3,6 positions on the benzoquinone ring appears to influence which activation mechanism predominates (Fig. 8B). For structures possessing small substituents at one or both sites, reduction occurs *via* a type I NTR activity with this mechanism unaffected by the presence of equivalent (e.g., hydrogen for DZQ, methyl for MeDZQ) or divergent (e.g., hydroxymethyl and methyl in RH1, aziridine and hydrogen in TZQ) groupings at the 3,6 positions. Such small side chains may aid type I NTR/compound interaction or help the prodrug (or its activated products) gain access to the site(s) of action. In contrast, the presence of bulkier groups (e.g., aziridines for ABQ22) at both sites results in CPR activation. Why this mechanism operates to reduce this particular compound is open to speculation but it may reflect a steric hindrance effect such that the four aziridine groups found in ABQ22 preclude TbNTR from interacting with and reducing its quinone carbonyl groups while facilitating associations with TbCPR isoforms. Alternatively, this difference could be due to subcellular localisation such that ABQ22 may be readily transported into the *T*. *brucei* endoplasmic reticulum, the organelle where the TbCPRs are believed to reside, but unable to gain access to the parasite's mitochondrion, the site where TbNTR is found (Wilkinson *et al*., 2008).

Following protonation of the aziridinyl nitrogen, the resultant electrophilic intermediate undergoes nucleophilic attack leading to opening of the heterocyclic ring, formation of an aliphatic amine then alkylation of the target (Fig. 8A) (Hargreaves *et al*., 2000). If the targeting compound contains multiple aziridinyl groups, as is the case for the ABQs tested here, and the nucleophile is DNA, then two nucleobases within the nucleic acid double helix become covalently crosslinked. In many eukaryotes the SNM1 (also known as PSO2) nuclease plays a key role in resolving such DNA damage, with cells lacking this activity being specifically and highly susceptible to ICL inducing agents (Cattell *et al*., 2010). By phenotypically screening a selected set of trypanocidal ABQs against a *T*. *brucei snm1* null mutant line, we demonstrated that trypanosomes lacking this DNA repair enzyme were more susceptible to NTR and CPR activated prodrugs relative to wild type. Moreover, in parasites where TbSNM1 was absent but the activation mechanism (either TbNTR or TbCPR) is elevated, this susceptibility phenotype was exacerbated, providing a direct link between the two oxidoreductase activities and DNA damage. Extending these assays to investigate the trypanocidal action of ABQs that do not undergo NTR or CPR activation, revealed that these do not promote a form of DNA damage that can be repaired through an SNM1‐dependent pathway. It is plausible that these agents may still function as monofunctional or bifunctional DNA alkylating agents, leading to formation of a single site adduct or intrastrand crosslink or could act as modifiers of other biological molecules *via* their aziridinyl or quinone ring structures.

The continuous *in vitro* culturing of bloodstream form *T*. *brucei* in the presence of RH1 generated a parasite line that displayed cross‐resistance to a range of NTR‐activated prodrugs. Analysis of clones revealed that these cells had reduced Tb*ntr* expression. Additionally, lower mRNA levels were also noted for other open reading frames across the ∼200 kb Tb*ntr*‐containing polycistron; however, this effect did not extend to the surrounding transcriptional units. In contrast to trypanosomes selected for resistance towards other NTR activated prodrugs such as nifurtimox or fexinidazole (Wilkinson *et al*., 2008; Wyllie *et al*., 2016), the copy number of all tested open reading frames in the Tb*ntr*‐containing transcriptional unit were equivalent to wild type, indicating that the observed reduction in gene expression was not due to a large (>10 kb) DNA deletion event; the ‘strandswitch’ region, areas on the trypanosomal chromosome postulated to be regions where polycistronic transcription is initiated (Imboden *et al*., 1987; Muhich and Boothroyd, 1988), between the Tb*ntr*‐containing and adjacent transcriptional units is ∼6 kb. Although the exact molecular basis that leads to RH1 resistance has yet to be deciphered, it is plausible that the transcription initiation site of one of the two Tb*ntr*‐containing polycistrons (*T*. *brucei* has a diploid nuclear genome) may have been compromised resulting in the reduction in mRNA levels.

We have demonstrated that ABQs display significant growth inhibitory activities against *T*. *brucei*, *T*. *cruzi* and *L*. *major*, with two compounds (RH1 and TZQ) exhibiting high potency (EC_50_ values < 100 nM) against all three pathogens. However, this is accompanied by toxicity against macrophage (‐like) mammalian cells, a trait previously noted in primary mouse splenocytes (Miliukiene *et al*., 2014). This unwanted activity may be because such immune cells have the ability to constitutively express or to upregulate quinone oxidoreductases including NQO1 and NQO1‐independent activities that promote oxidative stress and/or DNA damage (Tudor *et al*., 2005; Potts‐Kant *et al*., 2012; Miliukiene *et al*., 2014). Although these cytotoxicity issues may preclude the use of these ABQs as therapies for the treatment of systemic infections, understanding how they mediate their trypanocidal activities can inform drug development. By exploiting the chemical features that facilitate NTR or CPR specific activation such as those reported here, novel trypanocidal prodrugs could be generated. Following their activation, these compounds could be designed to release cytotoxic metabolites that affect a range of biological processes potentially targeting different biochemical pathways. Such agents would be best used in a polytherapy, as is the case with the anti‐HAT nifurtimox‐eflornithine combination therapy, in order to minimize potential resistance.

## Experimental procedures

### Compounds

The compounds used in this study were obtained from the following sources: The aziridinyl benzoquinones (DZQ, MeDZQ, AZQ, TZQ and ABQ3–4, 6–19, 22–34) (Table 5) were supplied by the Drug Synthesis and Chemistry Branch, Developmental Therapeutics Program, Division of Cancer Treatment and Diagnosis, National Cancer Institute, United States, while RH1 was donated by Prof Frank Guziec Jr (Southwestern University, TX). The chemical, physical and potential toxicological properties of each compound can be accessed *via* the PubChem database (http://pubchem.ncbi.nlm.nih.gov/) or OSIRIS Property Explorer software (http://www.organic-chemistry.org/prog/). Nifurtimox was obtained from Prof Simon Croft (London School of Hygiene and Tropical Medicine, UK), DFMO from Prof Mike Barrett (University of Glasgow, UK), mechlorethamine HCl from Stratech Scientific, while puromycin, blasticidin, phleomycin, hygromycin b and tetracycline were purchased from Melford Laboratories.

**Table 5:** Structure of aziridinyl 1,4‐benzoquinones used in this study.

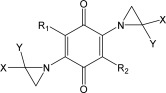			
Compound	Structure	Compound	Structure
ABQ1 (DZQ)	R_1_=R_2_=X=Y=H	ABQ18	R_1_=R_2_=NHCO(CH_2_)_5_CH_3_; X=Y=H
ABQ2 (MeDZQ)	R_1_=R_2_=CH_3_; X=Y=H	ABQ19	R_1_=R_2_=NHCOPhenyl; X=Y=H
ABQ3	R_1_=R_2_=Br; X=Y=H	ABQ20 (AZQ)	R_1_=R_2_=NHC(O)OCH_2_CH_3_; X=Y=H
ABQ4	R_1_=R_2_=F; X=Y=H	ABQ21 (TZQ)	R_1_=azirindyl; R_2_=X=Y=H
ABQ5 (RH1)	R_1_=CH_2_OH; R_2_=CH_3_; X=Y=H	ABQ22	R_1_=R_2_=azirindyl; X=Y=H
ABQ6	R_1_=R_2_=OCH_3_; X=Y=H	ABQ23	R_1_=R_2_=X=H;Y=CH_3_ (r orientation)
ABQ7	R_1_=R_2_=OCH_2_CH_3_; X=Y=H	ABQ24	R_1_=R_2_=X=H;Y=CH_3_ (s orientation)
ABQ8	R_1_=R_2_=O(CH_2_)_2_CH_3_; X=Y=H	ABQ25	R_1_=R_2_=Br; X=H; Y=CH_3_
ABQ9	R_1_=R_2_=NH_2_; X=Y=H	ABQ26	R_1_=R_2_=Br; X=Y=CH_3_
ABQ10	R_1_=R_2_=NHCH_3_; X=Y=H	ABQ27	R_1_=R_2_=Cl; X=H;Y=CH_3_
ABQ11	R_1_=R_2_=NHCH_2_CH_3_; X=Y=H	ABQ28	R_1_=R_2_=Cl; X=Y=CH_3_
ABQ12	R_1_=R_2_=NH(CH_2_)_6_CH_3_; X=Y=H	ABQ29	R_1_=R_2_=F; X=H;Y=CH_3_
ABQ13	R_1_=R_2_=NH(CH_3_)CH_3_; X=Y=H	ABQ30	R_1_=R_2_=F; X=Y=CH_3_
ABQ14	R_1_=morpholinyl; R_2_=F; X=Y=H	ABQ31	R_1_=R_2_=NHCOCH_3_; X=H; Y=CH_3_
ABQ15	R_1_=R_2_=NHCOCH_3_; X=Y=H	ABQ32	R_1_=R_2_=NHCOCH_3_; X=Y=CH_3_
ABQ16	R_1_=R_2_=NHCOCH_2_CH_3_; X=Y=H	ABQ33	R_1_=R_2_=NHCOCH_2_CH_3_; X=H;Y=CH_3_
ABQ17	R_1_=R_2_=NHCO(CH_2_)_2_CH_3_; X=Y=H	ABQ34	R_1_=R_2_=NHCOOCH_2_CH_3_; X=Y=CH_3_

All compounds tested satisfy Lipinski's Rule of 5 (http://pubchem.ncbi.nlm.nih.gov/) although all are predicted to have toxicity risks (http://www.organic-chemistry.org/prog/peo).

### Cell culturing

Bloodstream form *T*. *brucei brucei* (MITat 427 strain; clone 221a) cells were cultured in HMI‐9 media (Life Technologies) supplemented with 36 mM sodium bicarbonate, 0.014% (v/v) β‐mercaptoethanol and 10% (v/v) heat‐inactivated foetal calf serum (GE Healthcare) as described (Hirumi and Hirumi, 1989). A cell line (2T1) engineered to constitutively express the tetracycline repressor protein was grown in the modified HMI‐9 medium, supplemented with 1 μg ml^−1^ phleomycin (Alsford *et al*., 2005). *T*. *brucei* Tb*ntr* heterozygote lines were cultured in the presence of 10 μg ml^−1^ blasticidin while transformed 2T1 parasites over expressing Tb*ntr*, Tb*cpr2* or Tb*cpr3* were maintained in the modified HIM‐9 medium supplemented with 1 μg ml^−1^ phleomycin, 2.5 μg ml^−1^ hygromycin b and, where appropriate, 1 μg ml^−1^ tetracycline to induce protein expression (Hall *et al*., 2011). *T*. *brucei* lines expressing altered levels of the DNA repair enzyme TbSNM1 were grown in the presence 1 μg ml^−1^ phleomycin, 10 μg ml^−1^ blasticidin and/or 2 μg ml^−1^ puromycin, as appropriate (Sullivan *et al*., 2015).

The epimastigote form of *T*. *cruzi* was cultured in RPMI‐1640 medium (Lonza) supplemented with 5 g l^−1^ trypticase, 5 g l^−1^ HEPES pH 8.0, 20 mg l^−1^ haemin, 0.34 g l^−1^ sodium glutamate, 0.22 g l^−1^ sodium pyruvate, 2500 U l^−1^ penicillin, 0.25 g l^−1^ streptomycin (all Sigma‐Aldrich) and 10% (v/v) heat‐inactivated foetal calf serum (GE Healthcare) at 25°C (Kendall *et al*., 1990).

The promastigote form of *L*. *major* MHOM/IL/80/Friedlin was cultured at 25°C in modified M199 medium (Invitrogen) supplemented with 4 mM sodium bicarbonate, 40 mM HEPES pH 7.4, 0.1 mM adenine, 0.005% (w/v) haemin (all Sigma‐Aldrich), 25000 U l^−1^ penicillin, 25 mg l^−1^ streptomycin (GE Healthcare) and 10% (v/v) heat‐inactivated foetal bovine serum (GE Healthcare) (Kapler *et al*., 1990).

The human acute monocytic leukemia (THP‐1) cell line was grown at 37°C under a 5% (v/v) CO_2_ atmosphere in RPMI‐1640 medium (Lonza) supplemented with 2 mM pyruvate, 2 mM sodium glutamate, 2.5 U ml^−1^ penicillin and 2.5 μg ml^−1^ streptomycin, 20 mM HEPES pH 7.4 and 10% (v/v) heat‐inactivated foetal calf serum (GE Healthcare). Differentiation of THP‐1 toward macrophage‐like cells was carried out using 20 ng ml^−1^ phorbol 12‐myristate 13‐acetate (Sigma‐Aldrich) (Rovera *et al*., 1979).

### Selective screening of the *T. brucei* RNAi library

Selection of the *T*. *brucei* RNAi library was performed as described (Glover *et al*., 2015). Parasites (5 × 10^6^ cells) were treated for 24 hours with 1 μg ml^−1^ tetracycline to induce RNAi prior to addition of 30 nM RH1. Periodically, the cell density of the culture was determined and when appropriate, diluted in growth medium containing fresh RH1 (30 nM) and tetracycline (1 µg ml^−1^). Once an outgrowth population had been selected, genomic DNA was prepared for RNAi target identification (Alsford *et al*., 2012).

### Selection of laboratory generated RH1‐resistant *T. brucei*

Wild type bloodstream form *T*. *brucei* seeded at 1 × 10^4^ cells ml^−1^, were cultured in the presence of sub‐lethal concentration of RH1 (5 nM). Over a 100 day period, the ABQ concentration in the culture media was increased in a stepwise manner reaching a final concentration of 40 nM. The resulting line (designated as RH1^R^) was cloned by limiting dilution in the presence of RH1 and the phenotype of two clones (RH1^RC1^ and RH1^RC2^) analysed.

### Parasite growth inhibition assays

All growth inhibition assays were carried out in a 96‐well plate format (ThermoScientific). Bloodstream form *T*. *brucei*, *L*. *major* promastigotes and *T*. *cruzi* epimastigotes parasites in the logarithmic phase of growth were seeded at 1 × 10^4^, 5 × 10^5^ and 5 × 10^5^ cells ml^−1^ respectively in 200 μl growth medium containing different concentrations of the compound under study. After incubation at 37°C for 3 days (*T*. *brucei*), 25°C for 6 days (*L*. *major*) or 25°C for 14 days (*T*. *cruzi*), resazurin (Sigma Aldrich) was added to each well at a final concentration of 12.5 µg ml^−1^ (or 2.5 µg per well). The plates were further incubated at 37°C for 8 hours (*T*. *brucei*), 25°C for 24 hours (*L*. *major*) or 25°C for 24–48 hours (*T*. *cruzi*) before measuring the fluorescence of each culture using a Gemini Fluorescent Plate reader (Molecular Devices) set at λ_EX_ = 530 nm and λ_EM_ = 585 nm with a filter cut off at 550 nm. The change in fluorescence resulting from the reduction of resazurin is proportional to the number of live cells. A compound's EC_50_ value was established using the non‐linear regression tool on GraphPad Prism (GraphPad Software) and the statistical significance of any differences in parasite susceptibilities assessed using the Student's *t* test calculator (GraphPad Software).

### Mammalian inhibition assays

Differentiated THP‐1 cells seeded at 2.5 × 10^4^ cells ml^−1^ were incubated for 3 days at 37°C in a 5% (v/v) CO_2_ atmosphere in 200 μl modified RMPI‐1640 growth medium containing various concentrations of the compound under study. Resazurin (2.5 µg per well) was added to each culture and the plates incubated for a further 8 hours before determining the fluorescence of each sample.

Experiments involving animals were approved by the Animal Welfare and Ethics Review Board at LSHTM and conducted under license in accordance with the Animals (Scientific Procedures) Act 1986 (UK Home Office Project Licence PPL70/6997). Peritoneal macrophages were isolated from BALB/c mice by lavage 24 hours after intraperitoneal injection of 2% (w/v) soluble starch (Sigma). Macrophages (40,000) in RPMI‐1640 medium (Lonza) supplemented with 10% (v/v) heat‐inactivated foetal calf serum (GE Healthcare) were adhered overnight at 37°C in a 5% (v/v) CO_2_ atmosphere onto a 16‐well chamber slide. Cells were then treated with various concentrations of the compound under study and the cultures incubated at 37°C up to 5 days under a 5% (v/v) CO_2_ atmosphere. Untreated controls were analysed in parallel on each slide. Macrophages were fixed in 100% (v/v) methanol, stained with 10% (w/v) Giemsa, visualized with Leica DMRA2 light microscope using a ×100 oil immersion objective and images captured using a Retiga EXi Fast 1394 digital camera. The number of cells per field of view was determined with a minimum of 10 fields examined for each drug treatment. The average number of cells per field of view was determined and expressed as a % relative to untreated controls. All treatments were performed in quadruplicate.

### Quantitative PCR (qPCR)

The copy number and mRNA levels of various genes were analysed by qPCR. Genomic DNA or total RNA was prepared from wild type or RH1‐resistant *T*. *brucei* using the DNeasy Blood and Tissue or RNeasy Extraction kits (Qiagen), respectively. cDNA was synthesized from total RNA using the Superscript VILO cDNA synthesis kit (Invitrogen). All qPCRs were performed in triplicate with the PerfeCTa qPCR FastMix kit (Quanta Biosciences) on a CFX96 Touch Real‐Time PCR Detection System (BioRad). Fluorescence data was collected using the CFX Manager Software (BioRad), the products analysed by a melt curve after the final cycle and normalized against telomerase reverse transcriptase (Tbtert; Tb11.01.10190) using the comparative C_T_ method (Schmittgen and Livak, 2008; Brenndorfer and Boshart, 2010).

### Cell cycle arrest assay

Wild type *T*. *brucei* (5 × 10^5^ ml^−1^) were cultured for 16 hours in the presence of mutagen (1 µg ml^−1^ phelomycin; 35 µM mechlorethamine; 100 nM RH1). Cells were washed in phosphate buffered saline (PBS), fixed in 2% (w/v) paraformaldehyde in PBS and washed once more in PBS. Aliquots of the cell suspension (∼10^5^ cells) were air‐dried onto microscope slides and the samples mounted in Vectashield containing 4, 6‐diamidino‐2‐phenylindole (Vector Laboratories). Images were captured using Leica DMRA2 epi‐fluorescent microscope fitted with a digital camera (Hamamatsu Photonics).
